# Semidirect Resin Composite Veneers in a Patient with Bruxism

**DOI:** 10.1155/2024/5572481

**Published:** 2024-03-22

**Authors:** Franciele Floriani, Natália Fiuza Coelho, Ludimila de Azevedo Linhares, Sheila Cristina Stolf, Guilherme Carpena Lopes

**Affiliations:** ^1^University of Iowa, Prosthodontics Department, Iowa, USA; ^2^Federal University of Santa Catarina, Florianópolis, Brazil; ^3^Department of Operative Dentistry, Federal University of Santa Catarina, Florianópolis, Brazil

## Abstract

This case report details the esthetic rehabilitation of a 32-year-old male patient suffering from sleep bruxism, primarily manifesting as a fracture and significant loss of tooth structure in the anterior maxillary central incisors. To address these concerns, the patient underwent a restorative treatment involving the application of semidirect resin composite veneers on the maxillary incisors and direct resin composite restoration on the incisal regions of the maxillary canines. This approach not only restored the functional integrity of the teeth but also significantly enhanced the patient's esthetic appearance.

## 1. Introduction

Sleep bruxism (SB) notably compromises harmonic smile lines due to the presence of incisal wear [1]. As defined by the American Academy of Sleep Medicine (AASM), SB is characterized as repetitive jaw muscle activity, manifesting in the clenching or grinding of teeth and/or bracing or thrusting of the mandible [2]. Furthermore, the international consensus on bruxism assessment describes SB as masticatory muscle activity during sleep, characterized as rhythmic (phasic) or nonrhythmic (tonic), and distinct from movement or sleep disorders in otherwise healthy individuals [3]. A clinical diagnosis of SB involves a comprehensive assessment, including self-awareness, interviews with a sleep partner, investigations of masticatory muscle fatigue and pain, tooth wear, polysomnography (PSG), and electromyographic and electrocardiographic data, typically recorded by the Bruxoff® device [2, 4].

Tooth wear, often attributed to bruxism, also results from other factors important in differential diagnosis [5]. Enamel wear, particularly in the incisal and cuspal areas, can progress to dentin exposure [6]. This wear is influenced by enamel's microhardness, density, mineral composition, and protein distribution [7]. Masticatory forces, uniformly distributed along the enamel-dentine junction, contribute to this wear process [8]. Understanding these factors is crucial in distinguishing bruxism-related wear from other forms of dental erosion, attrition, or abrasion [9]. Accurate diagnosis is key to selecting the most appropriate treatment strategy, whether it be restorative interventions like semidirect veneers or other preventive or therapeutic approaches.

Prosthodontic rehabilitation of bruxism patients requires careful consideration of the patient's needs and available materials [10]. An appropriate restorative material must balance mechanical properties with esthetics [11]. Restorative options include direct and semidirect resin composite veneers, ceramic veneers, and full coverage crowns (ceramic crowns and metal-ceramic crowns) with correct diagnosis and excellent planning are essential for a predictable treatment procedure [12].

Ceramic veneers provide excellent esthetic performance, bonding, sealing capacity, and bond strength to resin cement [13, 14], resulting in greater longevity [15]. However, factors like tooth preparation, bonding technique, and patient self-care significantly influence their success [16–20]. The high cost and potential for fracture and wear on opposing tooth surfaces make ceramic veneers less feasible for some patients, leading to the popularization of alternatives like direct resin composite veneers [21, 22].

The physical, mechanical, and esthetic properties of resin composites derive from their components, such as bisphenol A-glycidyl methacrylate (Bis-GMA), urethane dimethacrylate (UDMA), triethylene glycol dimethacrylate (TEGDMA), and 2-hydroxyethyl methacrylate (HEMA), which contribute to the desired viscosity [23]. The primary concern with resin composites is their optical properties, crucial for achieving natural-looking restorations [24]. Value, chroma, and hue are fundamental color dimensions in dental practice. A tooth's appearance is influenced by interactions with light, including absorption, transmission, reflection, refraction, and scattering [25].

Semidirect resin composite veneers, fabricated extraorally on a stone model or special impression material, offer advantages over direct resin composite veneers, such as reduced polymerization shrinkage stress, excellent marginal adaptation, superior finishing/polishing, enhanced tooth detail reproduction, and precise interproximal contact points [19]. The longevity of semidirect veneers relies on selecting suitable surface bonding treatments, adhesive systems, and resin cement [26]. While their use in posterior teeth (onlays/inlays) is well-established [27], fewer cases have been reported for anterior teeth [12]. This clinical case report illustrates the application of the semidirect resin composite veneer technique in a patient with SB.

## 2. Case Report

A 32-year-old male, in good overall health with no systemic health issues, visited the dental clinic, voicing concerns about the esthetic aspects of his anterior teeth. Initial clinical examination revealed incisal tooth wear on the buccal surface of the maxillary incisors [23]. Notably, there was a fracture on the incisal edge of a central incisor's direct resin composite restoration, which fortunately did not extend to the mesial and distal surfaces. Through a comprehensive assessment, including the patient's self-reported questionnaire (AASM), polysomnography (PSG), and clinical examination, a diagnosis of sleep bruxism (SB) was confirmed [1, 2].

Following angle classification, occlusal examination indicated normal occlusion class 1 malocclusion, characterized by the mesiobuccal cusp of the upper first molar occluding with the buccal groove of the lower first molar [28, 29]. The occlusal surfaces of the posterior teeth were intact, with no signs of flattening, fractures, chipping, or looseness. However, canine-guided occlusion was flattened in the anterior maxillary teeth, with a fractured resin composite observed. There was no reduction or alteration in the vertical dimension of occlusion (VDO) [30].

**Figure 1:**
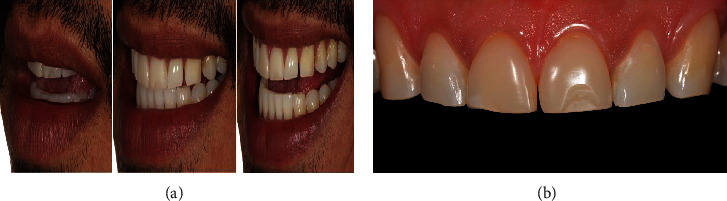
Series of photographs documenting the initial condition of the patient's maxillary anterior teeth. (a) The extraoral photograph taken during the first appointment highlights the relationship between the maxillary anterior teeth and the patient's lip. This lateral view distinctly reveals the absence of interproximal contact between the lateral incisor and the canine on the right side (elements #10 and #11, respectively), as well as noticeable dental wear, particularly in the cuspal and incisal areas, of the upper incisors and canines. (b) An intraoral photograph, providing a buccal view of the maxillary anterior teeth, clearly shows the existing direct resin composite veneers on the central incisors (elements #8 and #9). Notably, these veneers lack a shiny surface, and there is a visible fracture on the incisal third of the right central incisor (element #9).

Initial photographs (Figure 1) and bite-wing X-rays were taken for clinical case planning. Oral hygiene instructions were provided during the first appointment, including teeth cleaning, fluoride application, and brushing/flossing techniques. The treatment plan focused on reducing bruxism activity with a reduction of masticatory muscle activity with full mouth guards with hard occlusal splints (nightguards), and esthetic restoration [1, 2]. After the esthetic treatment, full mouth guards were prescribed to reduce bruxism activity and temporomandibular joint symptoms, promoting relaxation of facial muscles and protecting the natural dentition against further wear [1, 2].

The esthetic restorative treatment plan offered three options (Table 1): (1) replacing old direct resin composite restorations with direct resin composite veneers for the maxillary central and lateral incisors; (2) replacing the old direct resin composite restorations with semidirect resin composite veneers for the same teeth; (3) using ceramic veneers for these teeth. Additionally, direct resin composite restorations on the incisal edges of the maxillary canines were proposed in all treatment planning options. The patient chose the second option, favoring the benefits of semidirect resin composite veneers, such as reduced polymerization shrinkage stress, excellent marginal adaptation, better finishing and polishing, enhanced tooth detail reproduction, and precise interproximal contact points [10, 11]. This choice was influenced by the patient's parafunctional habits, with semidirect resin composite veneers deemed suitable for long-term prognosis. Ceramic veneers, though an option, were considered less ideal due to their higher cost, brittleness, and potential to cause wear on opposing tooth surfaces [27].

**Figure 2:**
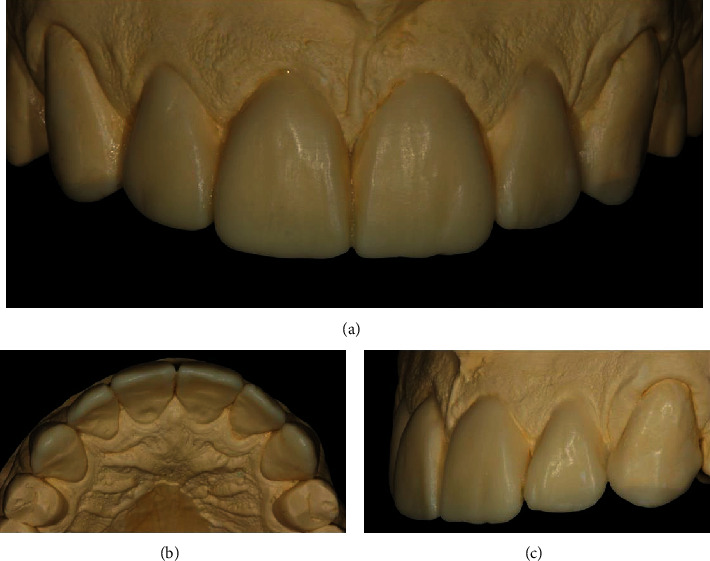
Diagnostic wax-up process conducted on a plaster model encompassing the canine to the contralateral canine (elements #6 to #11) aimed at restoring esthetics and dental anatomy. (a) The facial view of the wax-up model demonstrates the reestablished dental anatomy, providing a clear visualization of the proposed esthetic enhancements to the anterior teeth. (b) The incisal view of the model offers a detailed perspective of the reconstructed incisal areas of the teeth, highlighting the meticulous attention to detail in recreating natural tooth contours. (c) The left-side view of the wax-up model displays the newly designed shapes of the teeth, showcasing the proposed changes from a lateral perspective, which are crucial for achieving a harmonious dental appearance.

**Figure 3:**
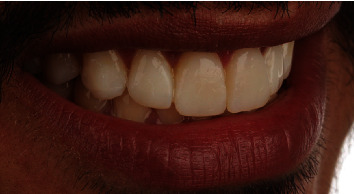
Smile view of mock-up procedure after being adjusted according to interocclusal space, patient's esthetic needs, and clinical evaluation.

To create stone models, upper and lower impressions were taken with polyvinyl siloxane material (Express XT, 3M Oral Care, St. Paul, MN, USA). The length of the maxillary incisors was established based on the patient's dental and facial proportions, and a diagnostic wax-up was performed on stone models of the maxillary canines to the contralateral canines (Figure 2). After creating the diagnostic wax-up, an esthetic mock-up was prepared with bis-acrylic self-cure temporary material (color A1, Structur 2, VOCO, Cuxhaven, Germany), which was applied inside the polyvinyl siloxane (PVS) guide, positioned correctly for 1.5 minutes, and then removed from the oral cavity. This mock-up provided a predictive preview of the potential esthetic changes posttreatment [31]. Changes were made to the mock-up, considering the patients' and dentists' esthetic perceptions (Figure 3).

**Figure 4:**
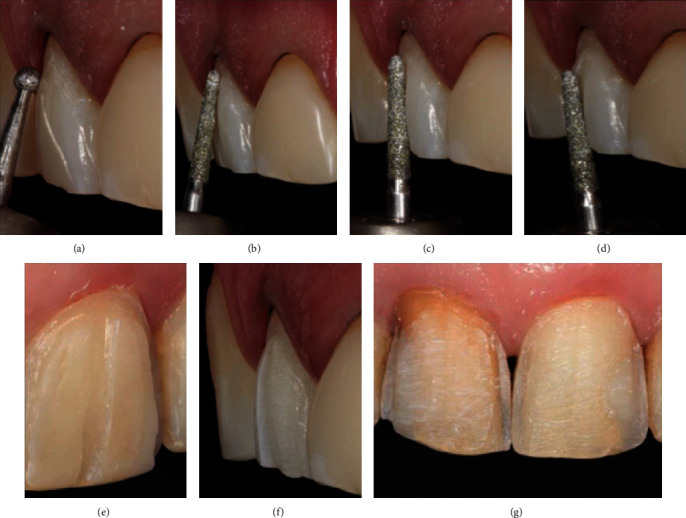
(a) Tooth preparation of the cervical with a spherical diamond bur at half depth and angled at 45°. (b) Longitudinal grooves in the cervical third were made with half of the active bur. (c) Longitudinal grooves in the middle third were made with half of the active bur. (d, e) Longitudinal grooves in the incisal third were made with half of the active bur. (f) Bur-prepared for half of the vestibular surface. (g) Bur-prepared of the vestibular surface; margins were refined with thin and extrafine diamond burs.

After mock-up approval, an impression was taken with polyvinyl siloxane (PVS) putty/light-body (Express XT, 3M Oral Care, St. Paul, MN, USA), and a new stone model was obtained. Complete field isolation was achieved using a rubber dam. Ideally, initial palatal photography or radiography would have been provided to justify the extensive preparations. The tooth preparation was carried out conservatively, above the old resin composite restorations, without removing additional dental structures or increasing the depth of preparation. The first stage of tooth preparation began at the cervical region with a spherical diamond bur (bur head *ø* = 0.1 mm, bur length 19.0 mm, #1012, KG Sorensen, Barueri, SP, Brazil) at half depth. It angled at 45° to the tooth-long axis (Figure 4(a)), followed by a round-end taper diamond bur (bur head *ø* = 1.6 mm, bur length 8.0 mm, #2135, KG Sorensen). Longitudinal grooves were created on longitudinal grooves on the buccal enamel with a half diameter of the round-end taper diamond bur, respecting the cervical (Figure 4(b)), middle (Figure 4(c)), and incisal (Figure 4(d)) inclination planes of the buccal surface tooth. The tooth was firstly bur-prepared for half of the buccal surface (Figures 4(e) and 4(f)) and then was extended to the other half of the buccal surface. The gingival and proximal margins were refined with fine and extrafine diamond burs (bur head *ø* = 1.6 mm, bur length 8.0 mm, #2135F, #2135FF, KG Sorensen), extending the tooth preparation to the dynamic visibly proximal areas (Figure 4(g)).

After the tooth was prepared, an impression was taken with polyvinyl siloxane (PVS), of medium viscosity (Express XT, 3M Oral Care), using the single-step technique. Two sizes of retraction cord (#000 and #00 Ultrapak; Ultradent Products Inc, South Jordan, Utah, USA) were inserted on the buccal and proximal areas of the marginal gingiva to free the gingival crest, and impressions were taken with the prepared teeth to obtain the stone models, in type IV stone (high strength, low expansion).

**Figure 5:**
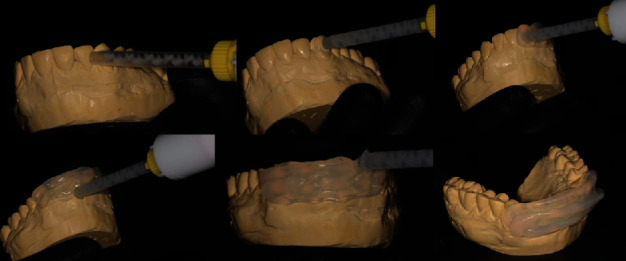
Step-by-step procedure of the manufacture of the vestibular guide with transparent silicone over the wax-up stone model.

**Figure 6:**
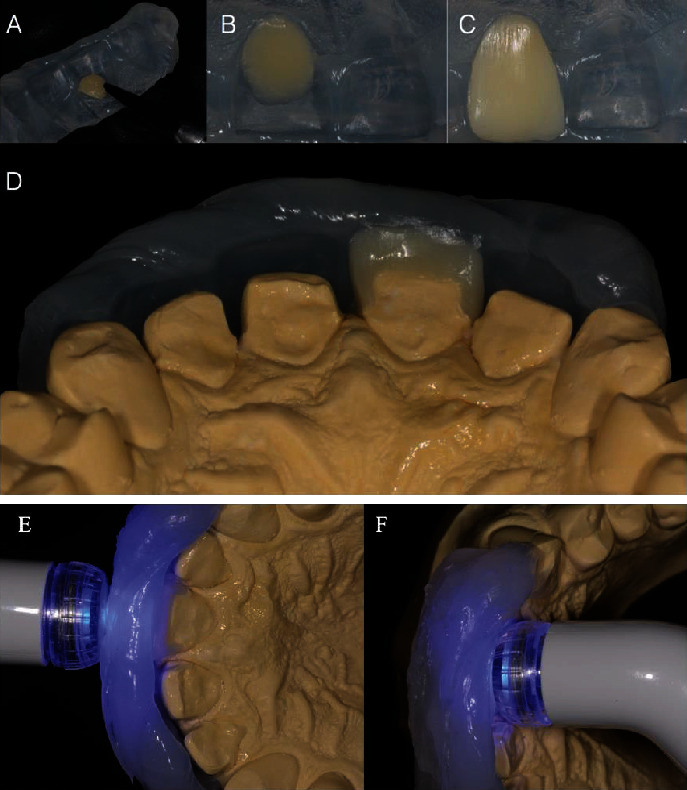
(a) Resin composite was adapted to the vestibular silicone guide. (b) Resin composite selected was color A3B for the cervical area. (c) The selected color of the composite resin is A1B for the middle third and incisal third. (d) Proximal surfaces were adjusted using the stone model and vestibular silicone guide. (e) Light-curing for 40 seconds on the buccal surface. (f) Light-curing for 40 seconds on the palatal surface.

**Figure 7:**
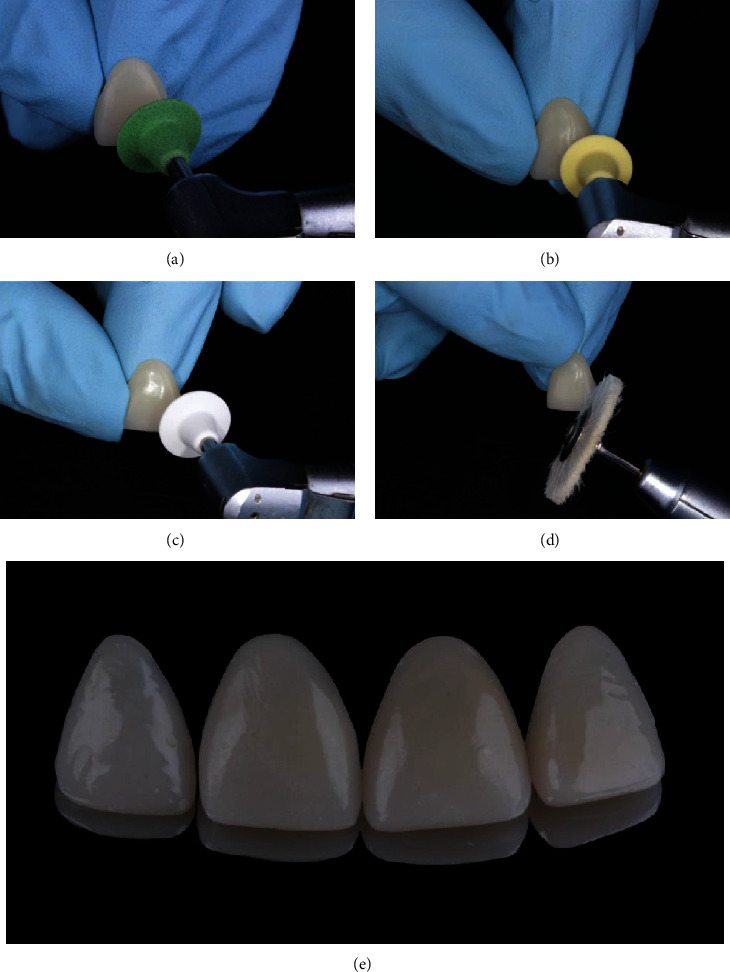
Finishing and polishing steps. (a) Abrasive rubber silicone discs with progressive reduction of abrasive size, starting with discs of medium abrasive size. (b) Fine abrasive size. (c) Superfine abrasive size. (d) Final polishing by the brushes and diamond paste. (e) Final aspect of semidirect resin composite veneers.

**Figure 8:**
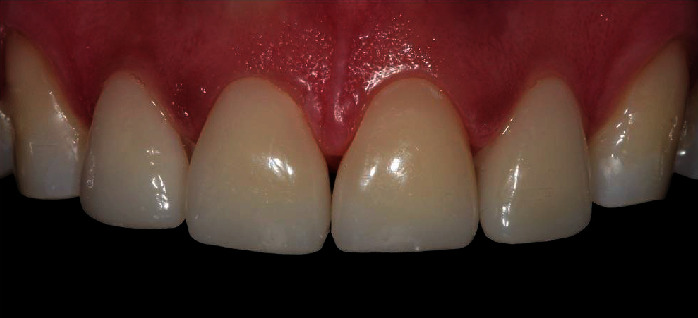
Marginal adaptation of semidirect resin composite veneers to the tooth preparations. Facial view. Note the presence of gingival inflammation on tooth #10. For this reason, a hemostatic agent with aluminum chloride was used to control bleeding and gingival fluid before cementation.

The semidirect resin composite veneers were made from a vestibular guide with transparent silicone (Panasil Tray Soft and Panasil Initial Contact Light; Kettenbach GmbH & Co, KG, Kettenbach GmBH & Co, Eschenburg, Germany) (Figure 5). The resin composite was adapted to the transparent silicone guide and cured with a LED light-curing unit (Radii, SDI, Victoria, Australia), with an output of 1,200 mW/cm^2^ for 20 seconds (Figure 6(a)). The colors of the resin composites selected were A3B (Filtek Z350 XT, 3M Oral Care) for the cervical third (Figure 6(b)) and A1B (Filtek Z350, 3M Oral Care) for the middle third and incisal third (Figures 6(c) and 6(d)). After light-curing (Figures 6(e) and 6(f)), the semidirect resin composite veneers were taken to the oven for complementary polymerization for 10 minutes in a Heraeus Siloc Oven (Heraeus Kulzer, South Bend, Indiana, USA) at 250 to 370°C. This handling method is safe and clean, saves time with a low heat load, and presents high process reliability due to a controlled temperature when compared to hot water [10–13]. The resin composite veneers of the proximal surfaces were adjusted using fine-grit abrasive discs (Sof-Lex Pop-On, 3M Oral Care), with the aid of the stone model and transparent silicone guide (Figure 6(d)). The semidirect resin composite veneers were finished with a decreasing sequence of Sof-Lex Pop-On discs (3M Oral Care) of medium, fine, and super-fine grits [10, 11]. Finishing/polishing was performed with a silicone polisher (Jiffy Polisher; Ultradent Products Inc, South Jordan, Utah, USA), starting with the discs of medium, fine, and then super-fine abrasive size (Figures 7(a)–7(c)), followed by the composite polishing wheel brushes (Figures 7(d) and 7(e)) [19]. Subsequently, to verify the adaptation of veneers to the dental structure, complete isolation with a rubber dam was performed. The try-in pastes (Variolink Esthetic Try-In, Ivoclar Vivadent, Schaan, Liechtenstein) were used to select the resin cement color (Figure 8).

**Figure 9:**
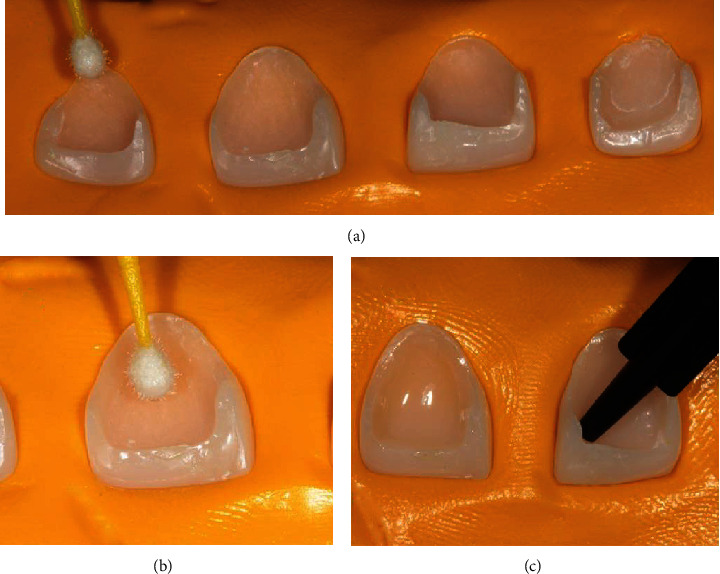
The semidirect composite resin veneers after sandblasting with 110 *μ*m aluminum oxide powder. Although not apparent in the image, note that they have been protected with PVS putty material to prevent sandblasting on the external surface. The semidirect composite resin veneer intaglios were prepared as follows: (a) Silane was applied with a small brush to the pretreated surfaces, and the material was allowed to react for 60 seconds. Subsequently, any remaining excess was dispersed with a strong stream of air. (b) The universal adhesive system was applied (AdheSE Universal, Ivoclar Vivadent, Schaan, Liechtenstein). (c) Dual-cure resin cement (Variolink Esthetic DC, Ivoclar Vivadent, Schaan, Liechtenstein) was applied to the internal surface of the semidirect composite resin veneers.

**Figure 10:**
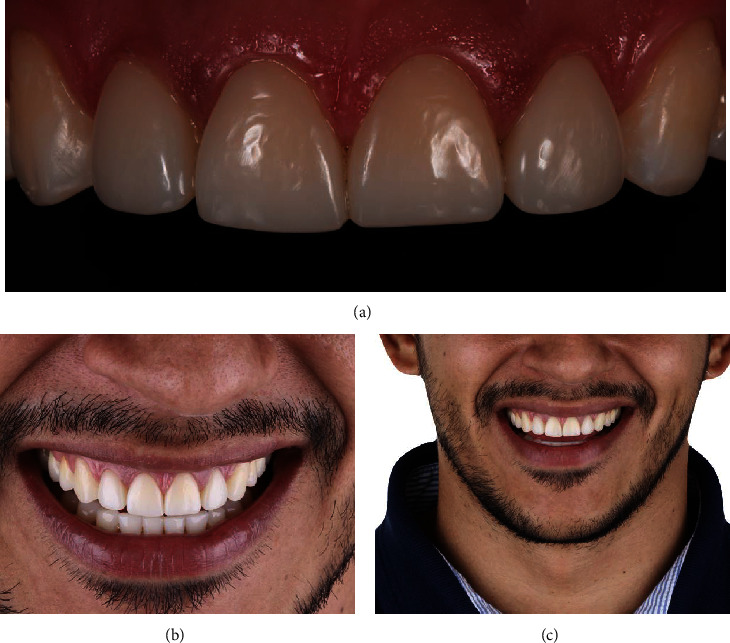
Final outcomes of the restorative treatment using intraoral and extraoral photographs. (a) The intraoral photography captures the semidirect resin composite veneers placed on the central and lateral incisors (teeth #7 to #10) and the direct resin composite restorations on the incisal areas of the canine teeth (teeth #6 and #11). This view highlights the restoration of dental anatomy, interproximal contact, and the shiny surface, effectively rejuvenating the appearance and function of the incisal area between the canine teeth. (b) An extraoral photograph showcases the harmonious integration of the semidirect resin composite veneers with the patient's lip and smile, emphasizing the esthetic success of the treatment. (c) Another extraoral photograph demonstrates the natural appearance of the semidirect resin composite veneers in the context of the patient's overall facial esthetics. Following the cementation of the semidirect resin composite veneers, to protect the esthetic treatment from the effects of sleep bruxism, the patient was provided with an occlusal full mouth guard.

The intaglio surfaces of the semidirect resin composite veneers were protected with polyvinyl siloxane (PVS) (3M™ Express™ STD, 3M Oral Care) to avoid sandblasting of the external surface (Figure 9). Sandblasting was performed with 110 *μ*m aluminum oxide powder, under a constant air pressure of 380 kPa, for around 10 to 15 seconds, at a 1.0 cm distance perpendicular from the nozzle to the surface [32]. This procedure was used to clean the surface and to increase its roughness, in order to enhance bonding by micromechanical interlocking [27]. Distilled water was then applied ultrasonically for 10 min, before applying silane (Monobond Plus, Ivoclar Vivadent, Schaan, Liechtenstein) for 60 seconds (Figure 9(a)) [27], followed by a layer adhesive system (AdheSE Universal, Ivoclar Vivadent, Schaan, Liechtenstein) without light-curing in this step, so there is not any risk of a thin layer, already polymerized, to interfere with restoration adaptation and protect the coated surface from light until applying the cement for seating, according to manufacturer instructions for veneers cementation protocol (Figure 9(b)) [33]. Phosphoric acid gel (37%) was applied to prepare teeth for 15 seconds with a subsequent rinse-off, and drying, before application of the universal adhesive system (AdheSE Universal, Ivoclar Vivadent) [33]. The adhesive was rubbed over the entire surface for 20 seconds, and an airstream was then applied for 10 seconds for solvent evaporation without light curing at this time [33]. A dual-cure resin cement (Variolink Esthetic DC, Ivoclar Vivadent) was applied to the intaglio of the semidirect resin composite veneers and positioned on the tooth (Figure 9(c)) [34]. Resin cement excess was removed with a disposable brush and dental floss, followed by a light-cure step using a LED light-curing unit (Radii, SDI, Victoria, Australia) of the adhesive system and resin cement for 40 seconds on each surface (buccal and palatal) [30]. Then, the resin cement excess was removed at the cervical margins with a scalpel blade (#12, Hu-Friedy, Chicago, USA) and with finishing strips (Epitex Finishing & Polishing Strips, GC America Inc., Chicago, USA) on interproximal surfaces [35]. Finally, the resin composite direct restorations were made on the upper canines to reestablish the canine guide (Figure 10).

## 3. Discussion

This case report is aimed at reducing bruxism activity and providing pain relief, while also discussing the esthetic treatment technique utilized. The semidirect resin composite veneer technique, chosen over direct resin composite restoration, offers improved contouring of proximal surfaces and interproximal contacts, superior wear and fracture resistance of the resin composite [19], and reduced chair time compared to ceramic veneers [36]. For this clinical case, the main appointment, encompassing tooth preparation, impression, and luting procedures, required approximately 50 minutes of chair time, followed by an additional 60 minutes dedicated to laboratory steps.

Regarding the esthetic rehabilitation of the central and lateral upper maxillary incisors in patients with bruxism, there is no consensus in the literature about the best materials and techniques [4, 37]. Studies have shown that ceramic veneers, while esthetically pleasing, are prone to fractures and debonding in patients with active bruxism [38]. Postesthetic treatment, the management of bruxism included the use of an occlusal full mouth guard to reduce bruxism activity and provide pain relief. Considering that 85% to 90% of the population may experience SB at some stage in their lives [11], it becomes essential to incorporate a full mouth guard in treatment planning with hard occlusal splints (nightguards) essential for safeguarding the esthetic restorations from the detrimental effects of bruxism [37].

In this clinical case report, through a comprehensive assessment, including the patient's self-reported questionnaire (AASM), polysomnography (PSG), and clinical examination, a diagnosis of sleep bruxism (SB) was confirmed [1, 2]. However, previous study evaluated factors associated with tooth wear other than bruxism, as a result, higher occlusal wear scores in the incisor and canine regions compared to the posterior region [23]. It was found that the overall progression in an 18-month follow-up period was slow, and, the need for future treatment may be based on such an evaluation of the progression of wear [23]. Thus, long-term follow-up is crucial to evaluate the success of treatment, especially in patients with SB or wear.

The success of the semidirect technique hinges on meticulous attention to the veneer's intaglio surface treatment, including chemical and mechanical steps, careful adhesive system selection, and the choice of resin cement [25]. Sandblasting with aluminum oxide, followed by silane-coupling agent application, optimizes the bonding interface between the resin composite veneer and the resin cement [32]. Universal adhesives containing functional monomers like 10-MDP can further enhance this bond [39]. Moreover, the semidirect approach obviates the need for provisional veneers, thereby avoiding potential issues related to provisional cement that could adversely affect the final bond strength and veneer adaptation [19]. This approach also allows for the use of dual-cure resin cement, ensuring a deeper cure and better optical properties of the final restoration [40].

The semidirect resin composite technique facilitates a laboratory polymerization process that significantly improves the mechanical properties of the resin composite compared to direct resin composite restorations. This additional polymerization, as prescribed by each manufacturer of the indirect resin composite [19, 41], ensures enhanced durability and performance.

Fahl and Ritter, in a review, provided an update on the direct-indirect composite veneer technique and showed that in the semidirect resin composite veneer technique, the selected composites are initially applied on the tooth using a layering approach [19]. However, in this clinical case, the choice of resin composite for semidirect veneers was a critical factor in achieving the desired esthetic outcomes. One of the newer commercial brands used was the 3M Filtek Universal Restorative (3M Oral Care), notable for its simplified shade guide system. This system, aligned with the Vita Shade Guide, offers eight color options and one universal opaque, facilitating accurate color matching [11]. The 3M Filtek Universal Restorative is a nanofiller resin composite, distinguished by its unique composition of filler materials. It includes a combination of nonagglomerated and nonaggregated silica particles (20 nm) and zirconia particles (4 to 11 nm), along with agglomerated ytterbium trifluoride filler (100 nm). This advanced composition provides enhanced physical properties, crucial for the durability and esthetics of the veneers [41]. In the context of semidirect veneers, the ability to accurately replicate the natural coloration and translucency of teeth is imperative for achieving seamless esthetic integration with the patient's natural dentition [42–44]. Hence, the selection of 3M Filtek Universal Restorative in this case not only facilitated an optimal esthetic match but also ensured that the restorative material met the functional demands of the treatment, an essential consideration for the long-term success of semidirect veneer.

In the clinical case presented, a flattened canine-guided occlusion was observed, along with a fractured old resin composite in the anterior maxillary teeth. Canine-guided occlusion, a form of mutually protected occlusion, ensures the disengagement of posterior teeth during lateral mandibular movements, thanks to the vertical and horizontal overlap of the canine teeth [42]. Given the importance of proper cuspid guidance in protecting the incisal edges of the anterior teeth from abrasion and abfractions [28, 29], direct resin composite restorations were applied to the incisal edges of the canine teeth (element #6) and the first premolars (element #11).

While our manuscript primarily focuses on semidirect resin composite veneers, it is pertinent to briefly compare these with other full-coverage options such as zirconia crowns and metal-ceramic restorations, particularly considering their application for bruxism patients [37]. Zirconia crowns represent a modern solution, offering exceptional strength and esthetics. Their high fracture toughness and excellent wear resistance make them highly suitable for bruxism cases where durability is a primary concern. Furthermore, recent advancements in zirconia materials have significantly improved their esthetic appeal, rendering them appropriate for anterior teeth restorations [45]. In contrast, metal-ceramic restorations have a long-standing history in dental practice, recognized for their dependable strength and longevity [46]. These restorations provide a balance between durability and esthetics, although their use in the anterior region may be somewhat limited due to esthetic considerations [46]. When contrasting these options with semidirect resin composite veneers, important factors to consider include the extent of tooth preparation required, esthetic demands, and the functional load the restoration will endure. While zirconia and metal-ceramic crowns offer robust solutions for areas subjected to high functional loads and situations necessitating significant structural reinforcement, semidirect resin composite veneers offer a more conservative approach [36]. They preserve a greater amount of natural tooth structure while providing adequate durability and esthetics, making them well-suited for less severe cases of bruxism [19].

A significant advantage of semidirect veneers over crowns, irrespective of the dental material used, lies in their repairability [19]. In the context of patients with bruxism, this feature becomes exceptionally beneficial. The ability to repair semidirect resin composite veneers as opposed to replacing a crown presents a practical and cost-effective advantage, especially important in managing the recurrent challenges posed by bruxism. This feature ensures longevity and cost-effectiveness of the treatment, addressing one of the major challenges in managing dental conditions associated with bruxism.

## 4. Conclusion

This clinical case underscores the efficacy of semidirect resin composite veneers as a conservative, esthetic, and functional treatment option, especially for patients grappling with the effects of SB. It also highlights the need for comprehensive diagnostic and treatment planning, taking into account the patient's specific dental and occlusal conditions, to achieve successful outcomes. This case contributes to the growing body of evidence supporting the use of semidirect veneers in restorative dentistry, particularly for patients with bruxism, and encourages further exploration and documentation in this area.

## Figures and Tables

**Table 1:** Advantages of treatment possibilities: direct resin composite veneers, semidirect resin composite veneers, and ceramic veneers.

Direct resin composite veneers	Semidirect resin composite veneers	Ceramic veneers
Increased mechanical properties in composite resins [15, 18]	Increased mechanical properties of the resin composite in the semidirect composite resin veneers, excellent strength, and adhesion to the tooth structure [18] Improvement in physical and mechanical properties of the restorative material through complementary polymerization [10, 11]	Brittle, prone to fracture, and can induce wear with opposing tooth's surface [22] More resistant than direct resin composite and semidirect composite resin veneers [27]

More economical than semidirect composite resin veneers and ceramic veneers	More expensive than direct resin composite, and less than ceramic	Expensive

Depends on the clinical skills	Better contouring of proximal contacts and occlusal contacts, improved wear resistance, and reduced polymerization contraction when compared to direct composite resin veneers [12]	Better finishing and final polishing [18]

One appointment	One or two appointments	At least three appointments

Excellent durability [14]	Esthetics, durability	Esthetics, durability [18]

Preservation of remaining tooth structure because no need for additional retention, thus involving minimal intervention [14]	Conservative treatment option	Conservative treatment option for anterior teeth presenting wear and fractures [6]

The final color appearance of a composite restoration depends on the composition of the composite itself, the composite's thickness according to the substrate's color underneath it, pigment amount, and type and layering technique [37] The color stability after aging depends on the composition of the resin composite itself [37]	Better optical properties than direct resin composite due to the types of light-curing resin composite [10, 11] The color stability after aging appears to be more stable than direct resin composite due to the improvement of the physical and mechanical properties of the materials by reducing the thermal expansion coefficient and polymerization shrinkage, providing radiopacity, and improving the handling and esthetics of materials [17, 18]. The shrinkage of the material has been continuously improved over time [17, 18]	The optical parameters, surface texture, and translucency were affected by chemical aging on the glass ceramics Vita Mark II (Vita Zahnfabrik, Germany) and IPS Empress CAD (Ivoclar Vivadent AG, Liechtenstein), lithium disilicate-based ceramics e.max CAD (Ivoclar Vivadent AG, Liechtenstein), Vita Suprinity (Vita Zahnfabrik, Germany), zirconia-based ceramic IPS e.max: ZirCAD LT (Ivoclar Vivadent AG, Liechtenstein), and IPS e.max ZirCAD MT Multi (Ivoclar Vivadent AG, Liechtenstein) [37] The self-adhesive dual-cure cement showed color stability comparable to the total-etch light-cure resin cement for the cementation of IPS e.max (Ivoclar Vivadent, AG, Liechtenstein) ceramic veneers. The color stability of both cements was superior to that of the self-adhesive self-cure cement [38] Ceramic and resin-cement systems affected the color stability after the aging of ceramic veneers [38]

## Additional Points

*Clinical Relevance*. Bruxism poses a significant challenge to oral health, leading to complications such as tooth wear, masticatory muscle pain, and the fracturing of resin composite restorations. Semidirect resin composite veneers offer a conservative yet effective clinical solution, particularly for the anterior teeth. Despite their efficacy, there is a noticeable paucity of clinical cases documented in the scientific literature exploring the use of semidirect resin composite veneers in anterior teeth among bruxism patients. This gap underscores the need for more comprehensive studies and reports to better understand and validate the effectiveness of this technique in such scenarios.
